# Free Will and the Brain Disease Model of Addiction: The Not So Seductive Allure of Neuroscience and Its Modest Impact on the Attribution of Free Will to People with an Addiction

**DOI:** 10.3389/fpsyg.2017.01850

**Published:** 2017-11-01

**Authors:** Eric Racine, Sebastian Sattler, Alice Escande

**Affiliations:** ^1^Neuroethics Research Unit, Institut de recherches cliniques de Montréal, Montréal, QC, Canada; ^2^Biomedical Ethics Unit, Division of Experimental Medicine, Department of Neurology and Neurosurgery, McGill University, Montréal, QC, Canada; ^3^Department of Medicine and Department of Social and Preventive Medicine, Université de Montréal, Montréal, QC, Canada; ^4^Institute for Sociology and Social Psychology, University of Cologne, Cologne, Germany; ^5^Cognitive Science Program, McGill University, Montréal, QC, Canada

**Keywords:** free will, neuroimaging, addiction, responsibility, stigma, neuroscience, ethics

## Abstract

Free will has been the object of debate in the context of addiction given that addiction could compromise an individual's ability to choose freely between alternative courses of action. Proponents of the brain-disease model of addiction have argued that a neuroscience perspective on addiction reduces the attribution of free will because it relocates the cause of the disorder to the brain rather than to the person, thereby diminishing the blame attributed to the person with an addiction. Others have worried that such displacement of free will attribution would make the person with a drug addiction less responsible. Using the paradigmatic literature on the seductive allure of neuroscience explanations, we tested whether neuroscience information diminishes attributions of free will in the context of addiction and whether respondent characteristics influence these attributions and modulate the effect of neuroscience information. We performed a large-scale, web-based experiment with 2,378 German participants to explore how attributions of free will in the context of addiction to either alcohol or cocaine are affected by: (1) a text with a neurobiological explanation of addiction, (2) a neuroimage showing effects of addiction on the brain, and (3) a combination of a text and a neuroimage, in comparison to a control group that received no information. Belief in free will was measured using the FAD-Plus scale and was, subsequent to factor analysis, separated into two factors: responsibility and volition. The investigated respondent characteristics included gender, age, education, self-reported knowledge of neuroscience, substance-use disorder (SUD), and having a friend with SUD. We found that attributions of volition (in the cocaine-subsample) were reduced in the text and neuroimage-treatment compared to the control group. However, respondent characteristics such as education and self-reported knowledge of neuroscience were associated with lower attributions of responsibility for both substances, and education was associated with lower attribution of volition for the alcohol sub-sample. Interaction analyses showed that knowledge of neuroscience was found to generally decrease attribution of responsibility. Further research on attribution of free will should consider the effects of context and respondent characteristics, which appeared surprisingly larger than those induced by experimental treatments.

## Introduction

Free will is a commonly referenced but nevertheless complex concept. It is used both in academic and public discourse to describe an ability to choose between alternative courses of action (Stillman et al., 2011; Baumeister and Monroe, 2014; Monroe et al., 2014; Racine et al., 2017). In the context of addiction, free will has been an object of debate and scrutiny, since addiction could compromise an individual's ability to choose freely (Levy, 2013). In the philosophical literature, free will is often considered an all-or-nothing property, and it has been criticized for not capturing a positive ability of the agent *per se*, since it is often defined as the opposite of determinism (Gert and Duggan, 1979). Research on belief in free will, which includes a body of literature distinct from the long tradition of philosophical scholarship on the topic, has brought more attention to free will as a psychological phenomenon, i.e., a belief or disposition that has behavioral and motivational effects and is thus amenable to psychological inquiry (Baumeister, 2008; Baumeister and Monroe, 2014). This research has now shown that belief in free will can fluctuate and that such fluctuations have implications. For example, belief in free will can be modulated by both personal characteristics (e.g., physiological desires, religious beliefs, political orientations, self-esteem) (Laurene et al., 2011; Carey and Paulhus, 2013; Ent and Baumeister, 2014) as well as contextual or interpersonal characteristics (e.g., prompts about causal determinism diminishing belief in free will, differences between beliefs about one's free will vs. attribution to others) (Stroessner and Green, 1990; Vohs and Schooler, 2008; Baumeister et al., 2009; Pronin and Kugler, 2010; Lynn et al., 2014; MacKenzie et al., 2014; Nahmias et al., 2014). Moreover, changes in belief in free will have been associated with a number of consequential implications on attitudes and behaviors. For example, reduced belief in free will has been associated with diminished self-control (Rigoni et al., 2012) and helping behavior (Krueger et al., 2014), as well as increased cheating (Vohs and Schooler, 2008), increased punishment responses (Krueger et al., 2014) and increased aggressive behavior (Krueger et al., 2014). Higher belief in free will has been associated with more positive attitudes and behaviors, including ethically or socially desirable behavior (e.g., higher belief in free will predicted better job performance, Stillman et al., 2010; MacKenzie et al., 2014). Obviously, these findings like others in psychology and cognitive science could be affected by failures to replicate findings (Open Science Collaboration, 2015; Ewusi-Boisvert and Racine, in press).

Exposure to visual and textual neuroscience explanations for human attitudes and behaviors is one possible modulator of belief in free will (Vohs and Schooler, 2008; Vohs and Baumeister, 2009; Nahmias et al., 2014; Shariff et al., 2014). In discussions about the brain disease model of addiction (see explanation below) and its implications for treatment and policies, the effect of neuroscience information on belief in free will could matter significantly. Neuroscience information has been claimed to reduce the stigma associated with addiction (Dackis and O'Brien, 2005) because beliefs about the free will of people, as well as the associated attributions of blame and personal responsibility, are lessened (Racine et al., 2015). Alternatively, neuroscience information has been claimed to increase stigma because decreased attributions of free will infantilize individuals with an addiction and portrays them as dangerous because they are perceived to lack some basic requirement for decision-making and self-control (Hammer et al., 2013; Racine et al., 2015). Interestingly, other literature on the seductive allure of textual neuroscience explanations (Weisberg et al., 2008) or neuroimaging evidence (McCabe and Castel, 2008) has investigated whether specific forms of neuroscience information could sway beliefs about a host of phenomena (e.g., ratings of the value of scientific reasoning; explanations of psychological phenomena). In the following section, we further describe how the literature on the brain disease model of addiction sets the stage for the importance of belief in free will on different aspects of addiction, while the literature on the seductive allure of neuroscience explanations proposes specific approaches through which this effect could be investigated.

### Belief in free will and the brain disease model of addiction

**Figure 1:**
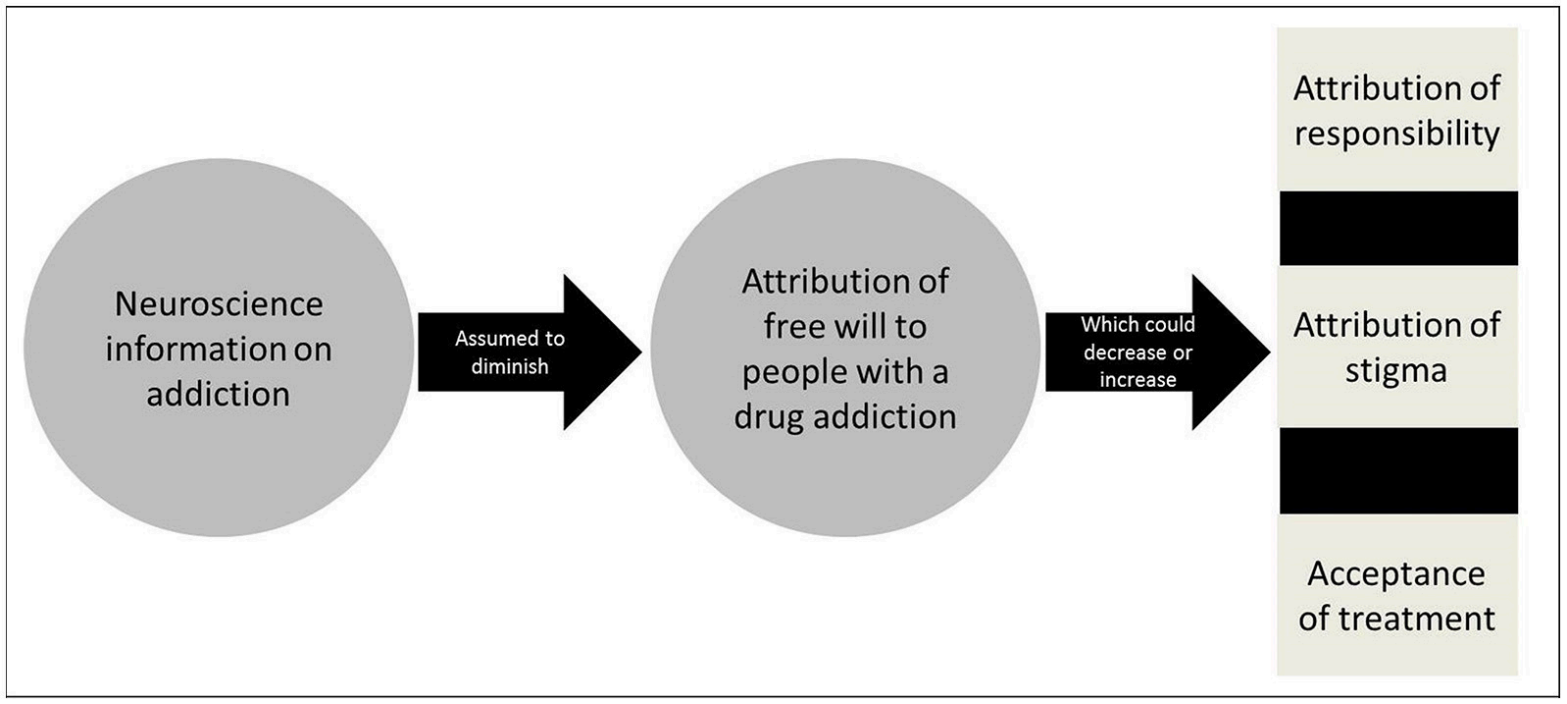
Impact of neuroscience information of attribution of free will. *Neuroscience information on addiction and attribution of free will:* Has now been generated as a result of the intensification of research activities on this topic in neuroscience. The implications of this research could be manifold, including for the basic understanding of the mechanisms of addiction, the development of treatment as well as prevention and policy (Dackis and O'Brien, 2005). *Belief in free will and attribution of responsibility in addiction:* Free will is often considered a pre-condition of attribution of responsibility for one's addiction and thus represents an important issue in philosophy and ethics (Sinnott-Amstrong, 2013). An emphasis on neuronal causes of addiction has been argued to remove, in part, the onus of responsibility of the individual because of their perceived or attributed lack of control or free will over their addiction (Hyman, 2007; Racine et al., 2015). In contrast to this brain disease view, the “moral model” of addiction stresses personal responsibility toward the addiction such that an individual with an addiction retains free will and personal responsibility for his/her condition (reviewed in Racine et al., 2015). As Holton and Berridge summarize, the tension between tenets of brain disease and moral views suggests that “[t]he two approaches are typically seen as quite incompatible. If addiction is a brain disease, then there is no role for willpower or self-control” (Holton and Berridge, 2013). *Belief in free will and attribution of stigma in addiction:* Belief in free will– often more or less clearly distinguished from beliefs in responsibility in the conceptual and empirical literature (Nadelhoffer et al., 2014) could relate to stigma against addiction and this represents an important concern in public health and an area of research in social psychology. Fierce debates have surfaced about the ability for biological information to diminish responsibility and related stigma in the form of blaming. On the one hand, attribution theory postulates that beliefs about someone's control over a situation or condition are related to the attribution of responsibility for that situation or condition (Martin et al., 2000; Corrigan et al., 2003). For example, if a person's condition is perceived as caused by that person's bad character, or “weak will”, such as in the case of peer influence, then the causes of the condition are perceived as being under that person's control and this individual is deemed responsible for his/her condition and therefore “blaming” could be seen as “warranted”. On the contrary, if a health condition is perceived as caused by a genetic abnormality, then the cause is seen as outside of that person's control and therefore the individual is not seen as responsible for the situation and “blame” would be an inappropriate response toward such a person. This effect has been unraveled in several studies (Corrigan et al., 2003; Dietrich et al., 2006; Sattler et al., 2017). On the other hand, and in spite of being common, the idea that biological information reduces attribution of free will, and thus diminishes certain types of stigma, remains contested with several studies reporting results to the contrary (Walker and Read, 2002; Phelan, 2005; Dietrich et al., 2006; Pescosolido, 2013). *Belief in free will and acceptance of treatment in addiction:* Belief in free will and related beliefs in self-control could support attitudes and behaviors associated with seeking (and complying with) treatment for addiction and this is an issue of importance in healthcare and treatment programs. Biological views on addiction would facilitate the uptake of treatment because the individual would no longer be considered at fault for his/her problem (at least not to the same extent) (Dackis and O'Brien, 2005). Also, blaming becomes futile for such a disease, thus paving the way, in principle, for greater acceptance of medical treatments (Gartner et al., 2012; Hall et al., 2015). However, stressing the biological nature of addiction has not necessarily been found to encourage treatment (Gartner et al., 2012) and could actually lead to fatalistic beliefs that undercut the motivation to follow treatment or beliefs in the control for the treatment of their condition (Vohs and Baumeister, 2009).

There have been debates about the impact of a brain disease model of addiction on a number of interwoven issues such as free will, responsibility, and stigma (notably blaming) (Levy, 2013; Hall et al., 2015; Racine et al., 2015). The core of the brain disease model of addiction is the “brain-hijack theory” (Leshner, 1997; Volkow and Li, 2005). It posits that addiction is a brain disease caused by a dysfunction of brain systems involved in reward and pleasure seeking. According to this view, a greater emphasis on the biological aspects of addiction is a gateway to greater social acceptance of people with an addiction (Dackis and O'Brien, 2005; Hyman, 2007). Indeed, this interest in the impact of neuroscience discourse on belief in free will can be understood not only because of its philosophical dimensions but also because of its practical relevance for a number of issues (see Figure 1).

However, the benefits of the brain disease model of addiction on relevant issues such as reduction of stigma and responsibility are disputed (Hall et al., 2015; Hart, 2017). Nonetheless, both those in favor of and those opposing the brain disease model of addiction appear to be in agreement about the actual existence of an effect of neuroscience information on belief in free will; otherwise, the debate would be moot (Holton and Berridge, 2013). Adding to this debate, brain disease models of psychiatric disorders such as addiction are considered to be gaining ground, sometimes at the expense of explanations based on psychological or social factors (Buchman et al., 2010).

### Belief in free will and the seductive allure of neuroscience information

Interestingly, a literature on the seductive allure of neuroscience explanations (Weisberg et al., 2008; Farah and Hook, 2013) and “neurorealism” (Racine et al., 2005; Rhodes, 2015) has tackled the issue of the actual impact of neuroscience on explanations of general psychological phenomena, and could shed light on the debate about the impact of the brain disease model of addiction on belief in free will. One influential study reported that (textual) neuroscience explanations have a “seductive allure” on naïve respondents because they increase the attributed value of a scientific explanation of psychological phenomena (e.g., mutual exclusivity, attentional blink) even if the neuroscience component of the explanation is irrelevant to what is being explained (Weisberg et al., 2008). This effect was found to be greater for poor explanations than for good explanations in the naïve respondents (general adult respondents, although the mean age for this group in this study was 20.1 years of age). Students in a graduate neuroscience course judged both the good and bad explanations as more satisfying when they contained irrelevant neuroscience verbiage. However, “experts” (a group of those who were either about to pursue, currently pursuing or already holding advanced degrees in cognitive neuroscience or cognitive psychology) were not swayed by the added neuroscience explanations (Weisberg et al., 2008).

Likewise, another landmark study suggested that neuroimaging evidence bears significant influence on the explanation of general psychological phenomena (McCabe and Castel, 2008). A first experiment showed that a companion neuroimage depicting the results, in comparison to a companion bar graph depicting the results, positively influenced the assessment of the description of the results and of the scientific reasoning in the article. A second experiment featured a complex topographical brain image, as the neuroimage could have been more persuasive in the first experiment simply because it was more complex. Neuroimages were found to increase the appreciation of the scientific reasoning in comparison to the topographical brain image. A third experiment featured a genuine news article from the BBC website summarizing data of a study published in *Nature* and discussing the potential for neuroimaging-based lie detection. The inclusion of a neuroimage increased values for the adequacy of the conclusion that brain imaging can be used as a lie detector, but not the evaluation of the adequacy of the title. The inclusion of criticism (for half of respondents) had no statistically significant effects for the assessment of the conclusion but diminished the assessment of the appropriateness of the title. Taken together, the Weisberg et al. and McCabe and Castel studies suggest that neuroscience information could have a seductive allure because neuroscience provides a convincing explanation for psychological phenomena. For example, neuroimages could provide “a physical basis for abstract cognitive processes, appealing to people's affinity for reductionist explanations of cognitive phenomena” (McCabe and Castel, 2008). These two studies launched further empirical investigations on the alleged “seductive allure” of neuroscience information (textual or neuroimaging). Two recent reviews have criticized these studies and their findings based on methodological grounds and on the lack of confirmation from other similar recent studies (Farah and Hook, 2013; Michael et al., 2013). Michael et al. reviewed data on the impact of neuroimages from a series of 10 experiments with 1,971 respondents, and found no statistically significant effects in contrast to McCabe and Castel's original findings. They also found no evidence that education or age moderated the influence of a neuroimage (Michael et al., 2013). The result that neuroimages have no persuasive explanatory power is somewhat puzzling because of previous debates, but the authors hypothesized that perhaps neuroimages are too technical to bring much additional value to the average reader. Another hypothesis is that people have become more skeptical about the explanatory power of neuroimages since the McCabe and Castel study (Michael et al., 2013). To test this latter hypothesis, the authors ran a series of five studies focused on the effects of textual information to replicate the effect found by Weisberg et al. They found more marked effects of textual neuroscience explanations. To explain this effect, the authors rightfully point out that, unlike McCabe and Castel, Weisberg et al. varied the quality of the scientific information and that McCabe and Castel added a neuroimage to a text already containing neuroscience explanations. Michael et al. (2013) propose that the effect of a neuroimage could be small or smaller when respondents have already been swayed by a neuroscience explanation (motivated reasoning), a question that they stress as important to address in the future. At this time, the debate about the actual effects of textual neuroscience or neuroimaging information is ongoing.

### Examining the impact of neuroscience explanations on belief in free will in the context of addiction

The present study seeks to contribute to both debates on the perception of free will in the context of addiction and to the seductive allure of neuroscience information. To shed some light on the debate about belief in free will in the context of the brain disease model of addiction, we used the paradigmatic approaches developed in the literature on the seductive allure of neuroscience. We designed an experimental study aimed at understanding the potential influence of neuroscience information (both textual and/or neuroimaging) on respondents' attribution of free will to a person with an addiction. The neuroscience information used in our study was taken from well-trusted and accessible websites (see section Instruments), and is thus information that might currently influence an individual's belief in free will outside our experiment. We chose to investigate addictions to alcohol and cocaine because they are amongst the most common addictions, and have varying effects on health and behavior (NIDA, 2011^1^). These substances also vary in their perceived addictiveness and potentially impact free will differently (Jasinska et al., 2014). For example, cocaine, an illicit drug, might be seen as leading to stronger addiction than a drug like alcohol, which is perceived as less addictive and more socially acceptable and thus induces different reactions and judgments (Cunningham et al., 1993; Schomerus et al., 2010; Sorsdahl et al., 2012; Sattler et al., 2017). Specifying the drugs allowed us to make the questions in the survey less abstract and more comprehensible to the reader instead of asking generally for addiction to substances. It also provided an opportunity to explore the robustness of findings by choosing two substances with different psychological, physiological, social effects, and user types. Special attention was granted to respondent characteristics (e.g., gender, age, neuroscience literacy) and their interaction with effects associated with neuroscience information. These characteristics have not yet been investigated thoroughly so far in the literature, with a few exceptions (notably Michael et al., 2013). The focus on addiction and the effects of neuroscience information on free will provided an anchor in a context where there are heated discussions about the impact of the brain disease model of addiction. Based on the research reviewed above, we formulated three primary research questions (research questions 1–3) and two secondary questions (research questions 4–5) stemming from our study design and tackling gaps in the literature.

Research question 1: Does a textual neuroscience description of addiction diminish attributions of free will compared to a control group that received no such information?
Research question 2: Do neuroimages referring to addiction diminish attributions of free will compared to a control group?
Research question 3: Does a combination of a textual neuroscience description and a neuroimage referring to addiction yield the strongest diminishing effect on attributions of free will compared to a control group?
Research question 4: Do respondents with different characteristics (such as age or neuroscience literacy) attribute different levels of free will to people with addiction?
Research question 5: How do such respondent characteristics shape the effect of neuroscience information on attributions of free will?

## Methods

### Participants and study design

For our experimental web-based study, we used the “WiSo-Panel” (Göritz, 2014). This opt-in panel includes 11,517 German members from all walks of life. Members are registered with basic information such as their name, e-mail-address, date of birth, and sex. Thus, while participation is not anonymous, it is voluntary. At any time, respondents have the opportunity to ask the panel-operator to delete their responses and all respondent data. Personal data and responses are stored in different databases. Names and e-mail-addresses were not matched with responses. On the first page of the questionnaire, respondents were asked to give informed consent about participation and data usage consistent with Canadian research ethics guidance, the Tri-Council Policy Statement (TCPS2). Secure sockets layer (SSL) protocols were used to encrypt answers of the respondents while responding. The e-mailed survey request explained the topic of the survey, its length, the field work duration (1 week), and the voluntariness of participation, and also that an incentive of 10 loyalty points (worth 1 Euro) would be awarded upon completion—which is a usual payment for this type of study. When a panel member receives 50 loyalty points, they can request a transfer of the money to their bank account or donate the money to the panel. By offering this reward, we hoped to increase survey participation and data quality (Lavrakas, 2008; van Veen et al., 2016).

About one quarter (26.20%, equaling *N* = 3,018) of the panel members viewed the first page of the survey. Of these, 94.67% (*N* = 2,857) consented to participate in the study and 97.83% of them (*N* = 2,795) completed the survey. Overall, the panel has an average response rate of 22.5% and average completion rate of 80%, thus the rates we obtained are slightly higher than the average for studies conducted with this panel (cf. Göritz, 2014). To ensure that our experimental treatments could have an impact on the respondents, we excluded respondents that had too short exposure times to these treatments^2^. Considering their exclusion and the exclusion of cases with missing values of any investigated variable, our analysis was based on 2,378 cases.

Almost 60% of the respondents were female (see Table 1 for descriptive statistics). The average age was approximately 46 years and the average number of years in education, which was based on two questions of the German Microcensus (Statistische Ämter des Bundes und der Länder, 2013) was 15 years. Thus, compared to the general population, our sample consists of more females (52%, information based on the German Microcensus), younger individuals (mean age in the general population: 49 years), and those with a higher education (mean years in education in the general population: 13 years).

**Table 1:** Descriptive statistics.

	**Mean**	**Standard deviation**	**Min**	**Max**
**ALCOHOL-SUBSAMPLE (*****N*** = **1,209)**				
Female	−0.58	–	0	1
Age in years	46.53	14.23	16	90
Education in years	15.17	2.63	7	21
Knowledge about neuroscience	2.70	2.29	0	10
Alcohol substance use disorder (SUD)	0.08	–	0	1
Alcohol substance use disorder (SUD) among peers	0.65	–	0	1
Base-line reading speed (BLRS)	−0.02	0.73	−0.71	8.62
FW_RESPONSIBILITY_	0.00	1.00	−2.82	1.92
FW_VOLITION_	0.00	1.00	−1.78	3.81
**COCAINE-SUBSAMPLE (*****N*** = **1,169)**				
Female	0.57	–	0	1
Age in years	46.41	14.40	17	92
Education in years	15.17	2.58	8	21
Knowledge about neuroscience	2.83	2.42	0	10
Cocaine substance use disorder (SUD)	0.01	–	0	1
Cocaine substance use disorder (SUD) among peers	0.09	–	0	1
Base-line reading speed (BLRS)	−0.01	0.86	−0.71	10.72
FW_RESPONSIBILITY_	0.00	1.00	−2.63	1.77
FW_VOLITION_	0.00	1.00	−1.63	3.60

*N, Number of observations*.

### Ethics statement

The ethics committees of the Institut de recherches cliniques de Montréal and of McGill University approved the study. All participants provided informed consent about participation and data usage consistent with Canadian research ethics guidance, the Tri-Council Policy Statement (TCPS2).

### Instruments

A professional translator translated those instruments that were originally developed in English to German according to the procedure described by Brislin (1970). This was followed by a back-translation by another professional translator. Corrections were then made after discussing potential differences. To test whether respondents understood all the questions, items, and instructions correctly, we ran cognitive pretests (*N* = 7) with German participants (with various socio-demographic backgrounds) by using a think-aloud technique and probing questions, i.e., we encouraged the respondents to think aloud when answering the online-questionnaire and we thereby wanted identify, for example, questions which seemed to be vague or difficult to understand. The insights gained from these pretests were used to refine the instruments.

### Experimental treatment

To assess the influence of neuroscientific information on belief about free will of people with addiction, we provided the participants with information depicting addiction from a neuroscientific point of view. We used three treatments and one control group (see Table 2). The control group received no information. Participants in the *text-only-*treatment were asked to read thoroughly a brief text extracted from brainfacts.org (2011)^3^, a well trusted and accessible website supported by the Society of Neuroscience, providing a neuroscience explanation of addiction. The text displays a marked biological reductionist overtone. The text presented to participants in the *text and neuroimage*-treatment included an additional neuroimage related to the topic of addiction and the brain also taken from the Internet (from drugabuse.gov; Davis, 2007), from the website of the National Institute of Drug Abuse. Respondents in the *neuroimage-only*-treatment solely saw the neuroimage. Presenting the text and the neuroimage independently and together allowed us to test whether effects differed for the text alone, the picture alone, or their combination. Furthermore, the sample was randomly divided in two: one half received follow-up questions concerning belief in the free will (see below) of people with an addiction to alcohol, and the other half answered questions focusing on people with addiction to cocaine.

**Table 2:** Experimental design^a^.

**Treatment**	**Text**	**Neuroimage**	**Reading instruction**
Control			[blank]
Text-only	^●^		Please carefully read the following definition of “addiction”. The next page then contains related questions. Then, please push the forward button.
Text and neuroimage	^●^	^●^	Please carefully read the following definition of “addiction” and carefully look at the picture depicting humans' brains after drug exposure. The next page then contains related questions. Then, please push the forward button.
Neuroimage-only		^●^	Please carefully look at the picture depicting humans' brains after drug exposure. The next page then contains related questions. Then, please push the forward button.
Text^b^	What is Addiction? Addiction is a chronic brain disease that causes people to lose their ability to resist a craving, despite negative physical, personal, or social consequences. People seek out nicotine and alcohol, or engage in gambling, because it makes them feel good or lessen feelings of stress and sadness. Many abused drugs produce a pleasurable feeling by exciting cells in the brain's reward center. With repeated use, drugs can change the structure of the brain and its chemical makeup [*displayed for text and neuroimage-treatment only*: (see example in the figure below)]. But why can some people casually drink alcohol or smoke cigarettes, while others fight to kick the habit? Neuroscience research, both in human and animal studies, is helping scientists identify key factors that influence susceptibility to addiction, such as a person's genetic makeup, vulnerability to stress, and the age they start engaging in the behavior. Slowly but surely, new studies are unraveling clues about processes in the brain that influence the likelihood of drug relapse. Such insights may help improve rehabilitation programs and drive down the global cost of addiction.		
Neuroimage^c^	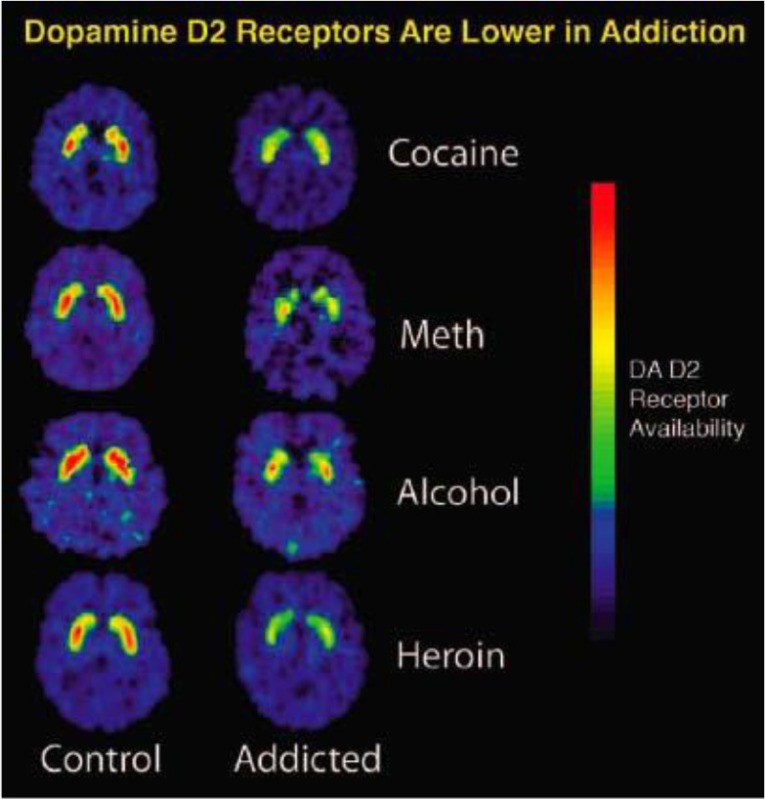		Effects of different drugs on the functioning of the brain: A comparison between the brains of non-addicts and addicts. The adjacent image shows that repeated exposure to drugs depletes the brain's dopamine receptors, which are critical for one's ability to experience pleasure and reward.

● *Indicates that this element was part of the experimental treatment*.

a *The sample was randomly assigned to these three experimental treatments or the control group displayed here. Furthermore, the sample was randomly divided into one group asked about the free will of people with addiction, while another group were similarly asked about cocaine*.

b *Adapted from: brainfacts.org (2011), a website supported by the Society of Neuroscience*.

c *Adapted from: from drugabuse.gov (Davis, 2007), the website of the National Institute of Drug Abuse*.

#### Belief in free will

After the experimental treatments (the control group received no prior information), we assessed belief in free will regarding people with a drug addiction. We therefore used seven adopted items (see Table 3) of the Free Will and Determinism (FAD-Plus) instrument (Paulhus and Carey, 2011), which is an enhancement of the preliminary FAD-4 version. As described above, the items were translated to German and back-translated to English, followed by a cognitive pretest. Participants were asked to think about people addicted to either alcohol or cocaine and under the influence of the respective substance and to rate these items on a 6-point scale ranging from “strongly disagree” [0] to “strongly agree” [5].

**Table 3:** Factor analysis and descriptive statistics for the Free Will (FAD-Plus) items.

**Items^a^**	**Alcohol-subsample (*****N*** = **1,209)**				**Cocaine-subsample (*****N*** = **1,119)**			
	**Factor loading**		**Mean**	**SD**	**Factor loading**		**Mean**	**SD**
	**F1**	**F2**			**F1**	**F2**		
1. They must take full responsibility for any bad choices they make.	−**0.84**	−0.03	3.29	1.48	−**0.84**	−0.04	3.39	1.48
2. In the case of criminals, they are totally responsible for the bad things they do.	−**0.86**	−0.02	3.33	1.53	−**0.87**	−0.00	3.46	1.52
3. They are always at fault for their bad behavior.	−**0.73**	−0.10	2.67	1.55	−**0.75**	−0.09	2.74	1.49
4. These people have complete control over the decisions they make.^*^	−0.28	−**0.79**	0.91	1.19	−0.21	−**0.79**	1.06	1.28
5. They can overcome any obstacles if they truly want to.	−0.16	−**0.64**	1.77	1.19	−0.06	−**0.68**	1.71	1.47
6. They have complete free will.	−0.19	−**0.59**	1.61	1.50	−0.09	−**0.71**	1.52	1.42
7. With the strength of their mind, they can always overcome their body's craving for [alcohol/cocaine] ^b^.^**^	−0.35	−**0.48**	2.05	1.58	−0.25	−**0.57**	1.81	1.47

*Factor loadings based on principal component factor analysis with an oblimin rotation (eigenvalues>1)—bold figures indicate the highest loading of an item; N, Number of observations; SD, Standard deviation; F1, FW_RESPONSIBILITY_; F2, FW_VOLITION_*.

a *Responses were assessed on a scale from “strongly disagree” (0) to “strongly agree” (5)*.

b *Displayed substance refers to the substance investigated for the respective subsamples for this item*.

* *p < 0.01*,

** *p < 0.001 (differences between the alcohol and the cocaine-subsamples based on t-Tests)*.

We used principal component factor analysis with oblique oblimin rotation (to allow the factors to correlate) to identify the dimensionality of the scale. The *Kaiser-Meyer-Olkin Measure* of 0.75 for the alcohol-subsample as well as for the cocaine-subsample indicated a good suitability of the data for structure detection. Based on this analysis, a two-factor solution was developed: for both subsamples, the items of the originally proposed one factor-solution were separated into a factor focusing on responsibility (FW_RESPONSIBILITY;_ items 1, 2, 3, with an *eigenvalue* above 2.83 for the alcohol subsample and 2.83 for the cocaine subsample) and one focusing on volition (FW_VOLITION;_ items 4, 5, 6, and 7, with an *eigenvalue* above 1.15 for the alcohol subsample and 1.33 for the cocaine subsample). Due to a satisfying internal consistency of the items within each of these factors (Cronbach‘s α for FW_RESPONSIBILITY_: 0.78 for the alcohol subsample and 0.77 for the cocaine subsample; and for FW_VOLITION_: 0.60 for the alcohol subsample and 0.67 for the cocaine subsample), we continued our analysis with these two factors.

The authors of the scale already mentioned that this scale assesses “assumptions about autonomy” and “declarations that people are responsible for their actions” (Paulhus and Carey, 2011, p. 97). The duality of the scale is also reflected in their remark that “free will beliefs are consistent with an internal locus of control but also include moral responsibility” (Paulhus and Carey, 2011, p. 99). Both factors are assumed to be conditions for free will (Lavazza and Inglese, 2015). For the following analysis, regression factor scores were used for each factor (the score 0 indicates an average attributed responsibility or volition, and 1 is the standard deviation), because usually some items are more important than others when explaining a certain construct. By using factor scores instead of unweighted sum scores, the different impacts of each item was accounted for (DiStefano et al., 2009). Descriptive results for this and the following two instruments are shown in Table 1. When describing the results we refer to the two factors as FW_RESPONSIBILITY_ and FW_VOLITION_, while we use the generic concept of free will to refer to literature that has not differentiated both factors.

#### Self-reported knowledge about neuroscience

Respondents described their overall knowledge about neuroscience by responding to the following item: “My knowledge of neuroscience in general is…” with an 11-point scale ranging from “very low” [0] to “very high” [10].

#### Substance use disorder (SUD)

An adopted version of the ultra-rapid screening for substance-use disorders (ASSIST-LITE) (Ali et al., 2013) was used to assess respondents' SUD of the two investigated substances, alcohol and cocaine. Based on this screening, respondents were either grouped as no alcohol-SUD (respectively no cocaine-SUD) [0] or as having a tendency toward an alcohol-SUD (respectively a cocaine-SUD) [1].

#### SUD among peers

Furthermore, SUD among peers was assessed by asking whether the respondents know anyone who is addicted to the two substances under investigation (cf. Sorsdahl et al., 2012). Respondents were either grouped as not knowing anyone with an alcohol-SUD (respectively a cocaine-SUD) [0] or as knowing peers with an alcohol-SUD (respectively a cocaine-SUD) [1].

### Statistical analyses

Our experimental data were analyzed for both subsamples (alcohol and cocaine) regarding the effects of the experimental treatments, self-reported knowledge about addiction, SUD, and SUD among peers on the FW_RESPONSIBILITY_ and the FW_VOLITION_. We used multivariate ordinary least squares (*OLS*) regression models and displayed standardized coefficients (*beta*) and *t*-values for the main effect models, while unstandardized coefficients (along with *t-*values) were used for the models with interactions effects. Furthermore, Wald post-estimation tests were used to explore statistical differences between the three experimental treatments. We also controlled our results for base-line reading speed (BLRS1) (see footnote 2).

## Results

### Experimental treatments (research questions 1, 2, and 3)

Table 4 shows to what extent the experimental treatments influenced respondents' judgments of free will (which was, subsequent to factor analysis, divided in two factors: responsibility (FW_RESPONSIBILITY_) and volition (FW_VOLITION_) for people with addiction to alcohol and cocaine. With respect to research questions 1 and 2, we found that respondents' judgments did not significantly differ statistically between the control group and the *text-only-*treatment, nor did they differ significantly between the control group and the *neuroimage-only*-treatment, thus research question 1 and research question 2 found negative answers. With respect to research question 3, we did find that a combination of text and neuroimage yielded a stronger diminishing effect, but only for Model 4. Respondents in the *text and neuroimage*-treatment attributed a moderately lower FW_VOLITION_ (*beta* = −0.07, *p* = 0.048) to people with an addiction to cocaine. Furthermore, a post-estimation Wald test showed that those in the *image-only-*treatment attributed a lower FW_RESPONSIBILITY_ to people with an addiction to alcohol compared to those in the *text-only-*treatment (*p* = 0.026).

**Table 4:** Linear regression models of the FW_RESPONSIBILITY_ and FW_VOLITION_ regarding people with addiction to alcohol or cocaine on experimental treatments and respondent characteristics.

	**Alcohol-subsample**				**Cocaine-subsample**			
	**Model 1 **FW**_**RESPONSIBILITY**_**		**Model 2 **FW**_**VOLITION**_**		**Model 3 **FW**_**RESPONSIBILITY**_**		**Model 4 **FW**_**VOLITION**_**	
	***beta***	***t-*value**	***beta***	***t*-value**	***beta***	***t*-value**	***beta***	***t*-value**
**EXPERIMENTAL TREATMENTS (REF**. = **CONTROL GROUP THAT RECEIVED NO ADDITIONAL INFORMATION)**								
Text-only	−0.06	−1.75	−0.02	−0.58	−0.05	−1.32	−0.06	−1.82
Text and neuroimage	0.00	0.05	0.05	1.36	−0.01	−0.24	−0.07^*^	−1.98
Neuroimage-only	0.02	0.53	0.02	0.51	−0.02	−0.44	−0.04	−1.17
**RESPONDENT CHARACTERISTICS**								
Female	0.02	0.55	−0.04	−1.37	−0.06^*^	−2.07	−0.04	−1.19
Age in years	0.03	1.08	−0.07^*^	−2.44	−0.04	−1.18	−0.07^*^	−2.41
Education in years	−0.09^**^	−3.03	−0.13^***^	−4.40	−0.10^***^	−3.36	−0.04	−1.40
Neuroscience-knowledge	−0.12^***^	−4.11	0.03	0.99	−0.06^*^	−2.08	0.06	1.87
SUD^a^	−0.02	−0.72	−0.03	−0.92	−0.04	−1.31	0.02	0.66
SUD among peers^a^	−0.06^*^	−2.02	0.00	−0.04	0.00	−0.08	0.04	1.29
BLRS	0.01	0.46	0.00	0.15	0.02	0.75	0.04	1.36
Intercept	0.63^**^	2.91	0.98^***^	4.48	0.90^***^	4.03	0.54^*^	2.41
Observations	1,209		1,209		1,169		1,169	
Adjusted *R*^2^	0.03		0.01		0.01		0.01	
*F*	4.89		2.81		2.63		2.13	
Probability > *F*	0.00		0.00		0.00		0.02	

*Beta, standardized coefficients*.

a *For the alcohol subsample, this measure refers to an SUD regarding alcohol, while it refers to SUD regarding cocaine for the cocaine subsample*.

* *p < 0.05*,

** *p < 0.01*,

*** *p < 0.001*.

### Respondent characteristics (research question 4)

To answer our fourth research question, we examined how several respondent characteristics related to FW_RESPONSIBILITY_ and FW_VOLITION_. *Gender:* In comparison to men, women rated that a person addicted to cocaine had lower FW_RESPONSIBILITY_ (*p* = 0.039). *Age:* Older respondents attributed a lower FW_VOLITION_ to people with addiction to alcohol (*p* = 0.015) as well as to those with cocaine addiction (*p* = 0.016). *Education:* A greater number of years of education was associated with lower attribution of FW_RESPONSIBILITY_ in the alcohol- (*p* = 0.003) and the cocaine-subsamples (*p* = 0.001) and with a lower attribution of FW_VOLITION_ for people with an addiction to alcohol (*p* < 0.001). *Self-reported neuroscience-knowledge:* Greater self-reported knowledge about neuroscience led to lower scores for FW_RESPONSIBILITY_ in the alcohol- (*p* < 0.001) as well as in the cocaine-subsamples (*p* = 0.038). *SUD:* An indication for either an alcohol or cocaine SUD had no statistically significant effect on participants' ratings. *Peer SUD:* Respondents who reported knowing somebody with alcohol SUD indicated a lower responsibility in people with addiction to alcohol (*p* = 0.044). *BLRS:* The BLRS did not bring any statistically significant effect on the respondents' evaluations.

### Interaction effects between experimental treatments and respondent characteristics (research question 5)

**Figure 2:**
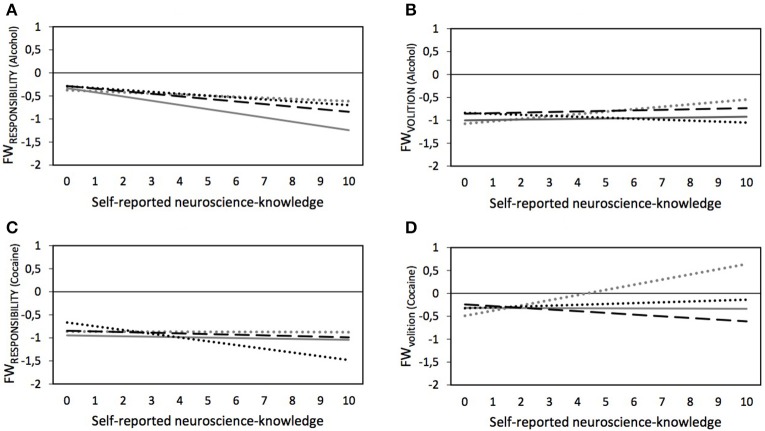
Predicted values for FW_RESPONSIBILITY_ and FW_VOLITION_ regarding people with addiction to alcohol **(A,C)** or cocaine **(B,D)** depending on experimental splits (… dotted gray lines, control group; — drawn gray lines, ”Text-only”; – – dashed black lines, “Text and neuroimage”; … dotted black lines, “Neuroimage-only”) and self-reported neuroscience-knowledge – based on Models 1 through 4 in Table 5, plotted for females without SUD, and subsample-specific average age, average education, and average BLRS.

In addition to the main effects analyses, we explored potential interaction effects between our experimental treatments and the respondent characteristics (research question 5), thus whether any of the respondent characteristics moderate the effects of the treatments. We found no statistically significant interaction effects for sex, gender, education, SUD, peer SUD^4^, and BRLS. While no statistically significant interaction effects occurred between the experimental treatments and neuroscience-knowledge with regard to the attributed FW_RESPONSIBILITY_ of people with addiction to alcohol (see Model 1 in Table 5 and Panel A in Figure 2), several interactions between these variables occurred for the three other dependent variables (FW_VOLITION−alcohol_, FW_RESPONSIBILITY−cocaine_, and FW_VOLITION−cocaine_) (see Models 2–4 in Table 5 and Panel B-D in Figure 2) which are described below.

**Table 5:** Linear regression models of the FW_RESPONSIBILITY_ and FW_VOLITION_ regarding people with addiction to alcohol or cocaine on experimental treatments and respondent characteristics.

	**Alcohol–subsample**				**Cocaine–subsample**			
	**Model 1 **FW**_**RESPONSIBILITY**_**		**Model 2 **FW**_**VOLITION**_**		**Model 3 **FW**_**RESPONSIBILITY**_**		**Model 4 **FW**_**VOLITION**_**	
	***B*-value**	***t*-value**	***B*-value**	***t*-value**	***B*-value**	***t*-value**	**B-value**	***t*-value**
**EXPERIMENTAL TREATMENTS (REF**. = **CONTROL GROUP THAT RECEIVED NO ADDITIONAL INFORMATION)**								
Text-only	0.04	0.34	0.07	0.57	−0.09	−0.69	0.17	1.37
Text and neuroimage	0.09	0.72	0.22	1.76	0.02	0.12	0.25	1.93
Neuroimage-only	0.09	0.72	0.24	1.95	0.19	1.50	0.17	1.29
**RESPONDENT CHARACTERISTICS**								
Female	0.04	0.62	−0.08	−1.39	−0.12^***^	−2.02	−0.07	−1.14
Age in years	0.00	1.07	−0.01^***^	−2.48	0.00	−1.26	0.00^*^	−2.15
Education in years	−0.03^**^	−3.06	−0.05^***^	−4.41	−0.04^***^	−3.48	−0.01	−1.22
Neuroscience-knowledge	−0.02	−0.96	0.05^*^	2.10	0.00	−0.06	0.11^***^	4.53
SUD^a^	−0.07	−0.70	−0.10	−0.94	−0.44	−1.36	0.21	0.65
SUD among peers^a^	−0.12^*^	−2.02	0.00	0.07	0.01	0.07	0.13	1.25
BLRS	0.02	0.46	0.01	0.19	0.02	0.68	0.05	1.52
**INTERACTIONS BETWEEN EXPERIMENTAL TREATMENTS AND NEUROSCIENCE-KNOWLEDGE (NK)**								
Text-only ^*^ NK	−0.07	−1.88	−0.04	−1.25	−0.01	−0.24	−0.12^***^	−3.37
Text and neuroimage ^*^ NK	−0.03	−0.92	−0.04	−1.16	−0.01	−0.35	−0.15^***^	−4.15
Neuroimage-only ^*^ NK	−0.02	−0.50	−0.07^*^	−2.12	−0.08^*^	−2.27	−0.09^**^	−2.69
Intercept	0.56^*^	2.50	0.88^***^	3.88	0.86^***^	3.67	0.24	−1.01
Observations	1,209		1,209		1,169		1,169	
Adjusted *R^2^*	0.03		0.01		0.01		0.01	
*F*	4.89		2.81		2.63		2.13	
Probability *> F*	0.00		0.00		0.00		0.02	

*B-Value, unstandardized coefficients; NK, Neuroscience-knowledge*.

a *For the alcohol subsample, this measure refers to an SUD regarding alcohol, while it refers to SUD regarding cocaine for the cocaine subsample*.

* *p < 0.05*,

** *p < 0.01*,

*** *p < 0.001*.

#### FW_VOLITION_ attributions regarding people with addiction to alcohol

As shown in Table 4, no statistically significant main effects for neuroscience knowledge and the experimental treatment were found on the perceived FW_VOLITION_ of people with addiction to alcohol. However, we also saw that neuroscience-knowledge had an effect in the control group (see Model 2 in Table 5). Specifically, increased neuroscience-knowledge slightly augmented (*p* = 0.036) the attributed FW_VOLITION_ of people with addiction to alcohol (see ascending dotted gray line in Panel B in Figure 2). However, increasing neuroscience-knowledge resulted in an opposite pattern in the *neuroimage-only*-treatment: here, increasing neuroscience-knowledge led to slightly lower perceived FW_VOLITION_ of people with addiction to alcohol (*p* = 0.035)^5^.

#### FW_VOLITION_ attributions regarding people with addiction to cocaine

No statistically significant overall effect for neuroscience-knowledge was found in the model on FW_VOLITION_ of people with addiction to cocaine without the interaction effects—as is visible in Table 4. Nonetheless, Model 4 in Table 5 (see also Panel D in Figure 2) show that, in the control group, increasing neuroscience-knowledge resulted in a higher attribution of FW_VOLITION_ to people with addiction to cocaine (*p* < 0.001). This effect of neuroscience-knowledge significantly differed for the other three experimental treatments, i.e., for the *text-only-*treatment (*p* = 0.001) and the *image-only-*treatment (*p* = 0.007) the respective lines were almost parallel to the x-axis, indicating no moderating effect of neuroscience-knowledge, and for the *text and neuroimage*-treatment an increase in neuroscience-knowledge slightly decreased the attributed FW_VOLITION_ of people with addiction to cocaine (*p* < 0.001).

#### FW_RESPONSIBILITY_ attributions regarding people with addiction to cocaine

In Table 4, we reported a negative main effect of neuroscience knowledge on the attributed FW_RESPONSIBILITY_ of people with an addiction to cocaine. As Model 3 in Table 5 and Panel C in Figure 2 show, by analyzing the interaction between neuroscience-knowledge and the experimental treatments, we found that increasing neuroscience-knowledge reduced FW_RESPONSIBILITY_ only for the *neuroimage-only*-treatment (*p* = 0.023). This interaction effect significantly differed from the interaction effect between neuroscience-knowledge and *text-only*-treatment (confirmed by a post-estimation Wald test, *p* = 0.036): the results show that no statistically significant effect of neuroscience-knowledge was found for the *text-only-*treatment. However, a post-estimation Wald test also indicated that for those respondents with the lowest value of neuroscience-knowledge, the attributed FW_RESPONSIBILITY_ was significantly higher in the *neuroimage-only*-treatment compared to the *text-only-*treatment (*p* = 0.030).

## Discussion

We embarked on an experimental study to test if the attribution of free will (which was, subsequent to factor analysis, divided in two factors: FW_VOLITION_ and FW_RESPONSIBLITY_) to people with a drug addiction was diminished by showing respondents a textual neuroscience description of addiction (research question 1), a neuroimage suggesting a biological basis for addiction (research question 2), or both (research question 3) in comparison to a control group. Both prompts were taken from publicly available sources to increase their relevance and ecological validity. To answer our secondary research questions, we also assessed how respondent characteristics affected free will attribution (research question 4) as well as how these characteristics interacted with the experimental treatments regarding these attributions (research question 5).

Besides a slightly lower FW_VOLITION_ attributed to people with a cocaine addiction after respondents were exposed to text and neuroimage information (research question 3), we found no significant main effects of textual information and/or neuroimaging in comparison to the control group (research question 1 and research question 2). We did find lower FW_RESPONSIBILITY_ attribution for people with alcohol addiction in the *neuroimage-only*-treatment in comparison to the *text-only-*treatment, but this appears as an isolated effect. In contrast to these negative results of the effects of textual information and neuroimaging, several respondent characteristics were more clearly but nevertheless weakly associated with attributions of free will (research question 4). In general, the largest effects were seen for education and knowledge about neuroscience, but these effects were still relatively small. Further analyses (research question 5) showed interaction effects between neuroscience-knowledge and the attribution of FW_VOLITION_ (for alcohol and cocaine) as well as FW_RESPONSIBILITY_ (for cocaine).

Overall, our results suggest that naturally occurring neuroscience information (as operationalized in this study) may have limited effects on attributions of FW_VOLITION_ and FW_RESPONSIBILITY_ to people with a drug addiction. However, we found various significant effects of respondent characteristics on these attributions. We discuss these findings (1) in light of ongoing controversies over the impact of neuroscience discourse on attribution of free will in the context of the debate on the seductive allure of neuroscience explanations, and (2) with respect to effects of respondent characteristics and how these bear on future research on the effects of seductive allure of neuroscience explanations, belief in free will, and the brain disease model of addiction. We acknowledge limitations to our study, including the possibility that respondents looked at the Internet or talked with others regarding addiction or neuroscience during the survey.

### Impact of neuroscience information on attribution of FW_VOLITION_ and FW_RESPONSIBILITY_

Our experimental study found only two effects: first, lower FW_RESPONSIBILITY_ attributions for people with cocaine addiction for combined textual and figurative neuroscience information compared to the control group and second, lower FW_RESPONSIBILITY_ attribution for people with alcohol addiction in the *neuroimage-only-*treatment in comparison to the *text-only-*treatment. In contrast, some have suggested that neuroscience information on addiction has effects because of the impact of the brain disease model of addiction (Dackis and O'Brien, 2005), and also because of the alleged significant effects of textual neuroscience explanations (Weisberg et al., 2008) and neuroimages (McCabe and Castel, 2008) on understandings of psychological phenomena. It has also been posited that neuroscience discourse directly undermines belief in free will in the context of addiction (Vohs and Baumeister, 2009). For example, Vohs and Baumeister write that, because willpower is influenced by psychosocial factors, the brain disease model of addiction could undermine self-control and responsibility because, historically, addiction has “acquired the connotation of loss of free will” (Vohs and Baumeister, 2009). Addiction is often understood as “a potent form of the belief that people cannot control and are not responsible for their actions” (Vohs and Baumeister, 2009). Our findings do not support these predictions and interpretations.

#### The inexistence of a general seductive allure of neuroscience explanations?

One possible interpretation is that neuroscience discourse and neuroimages simply do not carry the effects that both proponents and opponents of the brain-disease model of addiction have claimed. The literature on the seductive allure of neuroimaging, which offers a specific experimental context where the impact of neuroscience information has been investigated, seems to be pointing in that direction (Schweitzer and Saks, 2011; Schweitzer et al., 2011, 2013; Greene and Cahill, 2012; Gruber and Dickerson, 2012; Farah and Hook, 2013; Michael et al., 2013). Two reviews (Farah and Hook, 2013; Michael et al., 2013) and several other studies have now failed to replicate the original findings of McCabe and Castel (Gruber and Dickerson, 2012; Farah and Hook, 2013). One study has suggested that, based on the use of different types of neuroimaging information (e.g., inflated brain and whole brain images were more convincing), the perceived complexity of a neuroimage, rather than its familiarity or its resemblance to a real brain, could contribute to swaying beliefs about scientific explanations (Keehner et al., 2011). However, this study did not include a control group (e.g., no image or no brain image) and did not assess the initial effect reported by McCabe and Castel. In addition, a few studies have examined the impact of neuroimaging evidence on jurors, but the results are divided with some showing effects (Gurley and Marcus, 2008; McCabe et al., 2011; Ikeda et al., 2013) and others not (Schweitzer and Saks, 2011; Schweitzer et al., 2011; Greene and Cahill, 2012).

However, in contrast to the literature on the effects of neuroimages, the literature on the seductive allure of textual neuroscience explanations (Weisberg et al., 2008, 2015; Michael et al., 2013; Rhodes et al., 2014; Scurich and Shniderman, 2014; Fernandez-Duque et al., 2015; Rhodes, 2015) has so far evidenced a more robust effect, consistent with the original description of the phenomenon of neuro-realism in textual (print media) forms (Racine et al., 2005). Weisberg et al. have recently found that the length of the explanation could modulate the effects of the presence of textual neuroscience explanations, although this did not fully explain the effect. In contrast, the level of complexity (amount of jargon) in a textual neuroscience explanation does not appear to change the general effect (Weisberg et al., 2015). Interestingly, sample differences between undergraduate students and MTurk workers were noted, with the students generally attributing lower scores for neuroscience explanations, perhaps because the educational setting and its emphasis on critical thinking could be at stake (Weisberg et al., 2015). However, in one of the experiments in this study, students in psychology seemed particularly swayed by neuroscience explanations when the explanations were bad (Weisberg et al., 2015). The replication study by Fernandez-Duque et al. (2015), which included both textual and neuroimaging information found only effects for textual information, suggesting that the effect of neuroscience is conceptual (i.e., textual information on brain research best explains mental phenomena) and not pictorial (i.e., based on the representation of the brain *per se*). In contrast to this body of positive results, we found very limited statistically significant effects of neuroimaging in combination with the text (see results for research question 3).

Finally, consistent with our findings, recent studies on the impact of the brain disease model of addiction have not found strong effects of this model on attributions of stigma and blame, which should be reduced if attribution of free will is diminished by neuroscience information (Meurk et al., 2014b,c; Sattler et al., 2017). Alongside our own results, these recent findings suggest that the previous debate on the effects of the brain disease model of addiction on free will has potentially been overdone, at least in terms of the actual effects of neuroscience discourse on attribution of free will. The debate may have reflected the strong stances of the authors (Hall et al., 2015; Racine et al., 2015) and not necessarily of the general public, which does not seem to be swayed by neuroscience information.

#### Motivated reasoning interacting with the brain disease model of addiction?

Another possibility is that the effects of neuroscience information interact with pre-existing beliefs about the phenomenon at hand such as beliefs about the biological basis of addiction and its relationship to belief in free will. Scurich and Shniderman have found effects of motivated reasoning: there is a seductive allure of textual neuroscience information when the information confirms prior beliefs (Scurich and Shniderman, 2014). This finding is distinct from studies about motivated reasoning on the study of attributions of free will (Clark et al., 2014). Likewise, in the context of addiction, an individual already adhering to a brain-disease model of addiction could find neuroscience information more convincing while someone not adhering to a brain-disease model could find neuroscience information less compelling, and even repulsive. Since we did not survey pre-existing beliefs about the biological aspects of addiction, we cannot address such a possibility directly. However, other recent studies (Meurk et al., 2014b,c) have suggested that the general public does not strictly adhere to a brain-disease model of addiction. Accordingly, neuroscience discourse in the context of addiction may not have the effects that were initially predicted on stigma reduction (Dackis and O'Brien, 2005). It is possible that the understanding of addiction as brain disease and even as a disease may be a simplification and a form of reductionism not representative of public opinion, at least in Australia where that study was conducted (Meurk et al., 2014c). In sum, the existence of motivated reasoning cannot be ruled out.

#### The seductive allure of neuroscience explanations in high-stake, real-world settings?

In spite of the unlikeliness of a general phenomenon of seductive allure of neuroscience explanations, our study—and several others—leaves the possibility that higher-stake situations in real-world settings could elicit such a seductive allure effect. For example, the textual information we used, although found in a credible source, may not have sufficiently emphasized the biological basis of addiction to elicit effects. One could retort that the text was a piece of naturally occurring public discourse and that, even though more hyperbolic discourse could have greater effects, hyperboles could be easily criticized for their strong and artificial overtones. It is possible that neuroscience information, if it is emphasized or plays a more significant rhetorical role (e.g., to validate a discourse or provide additional confirmatory evidence) could play a more consequential role than found in our study. This would be consistent with the apparently significant influence that neuroimaging can have on patients and on clinical practices. For example, the use of imaging for back pain has increased dramatically (Chou et al., 2011), despite its debated clinical legitimacy, thus suggesting a significant influence of imaging on clinical practices. However, the discourse in which such imaging evidence is embedded (e.g., helps locate and visualize the pain) (Rhodes et al., 1999) could have considerable impact because it fits in a narrative where evidence is sought to confirm one's initial suspicion about the locus of pain (i.e., confirmation bias or “motivated reasoning”) (Scurich and Shniderman, 2014). Accordingly, in such a context, neuroimaging evidence could help to convince patients to side with medical opinions in favor of surgery or, in the context of addiction, neuroimaging could support certain types of biologically-grounded treatments for addiction to the detriment of more socially-grounded approaches (Hall, 2006; Dingel et al., 2011; Hall et al., 2015). We can thus speculate that in real-world contexts in which neuroimaging evidence is introduced, neuroscience information could play a greater role than found in more hypothetical settings. Furthermore, the fact that we partially found different effects for the substances at stake (cocaine and alcohol) could be an indication that the effects of a brain disease model of addiction need to be examined more specifically and with greater attention to different substance types (Buchman et al., 2010; Meurk et al., 2014a; Carter et al., 2016) or more generally to other phenomena that people can get addicted to and what these addictions imply.

In the future, it will be important to assess the effects of neuroscience information in naturally occurring discourse such as in the discourse of healthcare providers and its impact on patients or application in marketing approaches (e.g., promotional videos about addiction treatment on consumers using neuroimaging) in real social settings. For example, if the stakes of believing in neuroimaging are higher and aligned with other interests (e.g., seeking a diagnosis or insurance claims for addiction treatment), then perhaps they could play a larger role in attributions of free will than for those who have no such vested interest. In other words, the prior interests that one has in believing the credibility of neuroscience information may shape attitudes more than the information itself.

### Impact of respondent characteristics on the seductive allure of neuroscience explanations and attitudes toward free will in the context of addiction

Our study found that belief in free will is associated with some respondent characteristics such as education, self-reported knowledge about neuroscience, gender, or knowing someone with a SUD. From the standpoint of the literature on belief in free will, these results are somewhat surprising since there has been limited attention paid to the impact of respondent characteristics therein. Belief in free will has been found to persist across cultures (Sarkissian et al., 2010), but seems higher in religious individuals and higher among political conservatives than political liberals (Crescioni et al., 2016). However, a recent review of the literature of research on belief in free will found that most often respondent characteristics are only passively controlled for (e.g., to ensure there are no confounding effects of gender, level of education or age notably) and not actively investigated (Ewusi-Boisvert and Racine, in press). Also, many studies typically use much smaller samples than we did, with sample sizes most often below 250 participants with a few exceptions of studies with much greater sizes (Stroessner and Green, 1990; Nahmias et al., 2007, 2014; Mogi, 2014). These small sample sizes may have limited the ability to discover small to moderate effects of respondent characteristics. Additionally, the extensive use of samples of undergraduate students and other samples of young respondents (Ewusi-Boisvert and Racine, in press) may have prevented greater attention to important characteristics such as age and level of education (see below), given the homogeneity of these samples in these areas.

#### Respondent characteristics and the brain disease model of addiction

Much like our findings about the greater impact of respondent characteristics than neuroscience explanations on attribution of free will in addiction, the literature on the brain disease model of addiction suggests that respondent characteristics shape attitudes toward addiction to a greater extent than adherence to a brain disease model of addiction (Sattler et al., 2017). For example, women and older respondents have been identified as believing more that addiction is a disease (Meurk et al., 2014c). Being older and having more education (>15 years) was associated with less support for coerced treatment, and being older also predicted a lack of support for punishment by imprisonment (Meurk et al., 2014b). However, these last attitudes were not predicted by beliefs that addiction was a disease or a brain disease. Accordingly, like in our study, basic respondent characteristics appeared to be stronger predictors of attitudes toward addiction than adherence to the brain disease model in these studies (Meurk et al., 2014b,c).

Interestingly, a broader literature on attitudes toward stigma and blame in addiction also suggests that respondent characteristics play a role. We readily recognize the gap between the literature on belief in free will and the literature on stigma, but it is important to keep in mind that belief in free will, as operationalized in the FAD-Plus scale, measures responsibility, whereas a common measure of stigma, the attribution questionnaire (AQ), has for one of its explicit dimensions “blame,” i.e., that “people are responsible for and can control their mental illness” (Corrigan et al., 2003; Corrigan, 2012). In fact, there are possible parallels between findings based on the AQ (measuring stigma) and the FAD-Plus scale (measuring belief in free will). For example, we found that certain characteristics have been associated with less attribution of FW_RESPONSIBILITY_, such as being female (for cocaine addiction), more educated (for cocaine and alcohol), more self-reported knowledge of neuroscience (for cocaine and alcohol), reported knowledge of a SUD (for alcohol), have also been reported to lower blame toward people with an addiction (Sattler et al., 2017). As our study and the literature on the brain disease model of addiction and stigma associated with addiction suggest, factors such as respondent characteristics may have important effects on belief in free will. It is also possible that the effects of respondent characteristics were generated by the fact that we explored belief in free will in the specific context of addiction. Yet, at the same time, this would likely mean that other specific contexts (e.g., investigating the effects of belief in free will in everyday moral behavior, in the determination of criminal responsibility, in health behavior, etc.) could carry with them a series of context-sensitive beliefs and assumptions that are socially embedded or topic-specific, and where respondent characteristics play a role. Perhaps these characteristics could even have different roles in these different contexts. In this light, the interaction effects we found between belief in free will and self-reported knowledge about neuroscience (discussed below) could be a telling example of this potential social embeddedness of the seductive allure of neuroscience explanations.

#### Knowledge about neuroscience and the seductive allure of neuroscience explanations

In the context of research on the influence of neuroscience information, knowledge about neuroscience has been reported to have important effects in some studies while other respondent characteristics have typically not been found to lead to differences. For example, McCabe and Castel reported that the effects of neuroimaging evidence on the ranking of scientific explanations was particularly noteworthy for novice individuals but not for experts (McCabe and Castel, 2008). However, a replication study comprising 10 experiments (Michael et al., 2013) did not find differences regarding the effects of neuroimages related to education (and also age). Our analysis of interactions between the experimental treatments and the level of neuroscience-knowledge yields a complex picture that partially challenges the relationship found by McCabe and Castel but, more importantly, yields more specific questions to tackle.

Overall, our results suggest that increased neuroscience-knowledge results in lower attribution of FW_RESPONSIBILITY_ for people addicted to cocaine in the *neuroimage-only*-treatment. However, according to McCabe and Castel's study, one would perhaps expect neuroimaging to decrease attribution of FW_RESPONSIBILITY_ in novices but not in experts. However, we found that FW_RESPONSIBILITY_ was decreased in those who are more knowledgeable. This comparison assumes that neuroscience information undermines belief in free will in the context of addiction as suggested by Vohs and Baumeister (Vohs and Baumeister, 2009) and assumes also that what we measured as the level of neuroscience-knowledge can be mapped to concepts of novice and expert as deployed by McCabe and Castel. Indeed, most of our more knowledgeable participants remained nevertheless novices, see Table 1.

Despite these caveats, one possible interpretation of our findings is that greater emphasis on the biological underpinnings of addiction may lead to the belief, in the eyes of those with more neuroscience knowledge, that the person has less control over the addiction as predicted by proponents of the brain-disease model of addiction (Dackis and O'Brien, 2005). However, this effect is also the basis of the worries captured by critiques of the brain-disease model of addiction: namely that neuroscience information about addiction can actually exacerbate blame and stigma because the person with an addiction is seen as less able to take care of him/herself (Hammer et al., 2013; Szott, 2015). As a result of such beliefs, the person with an addiction can be considered passive and powerless, and relinquish his/her decision-making capacity to others such as healthcare professionals or state authorities (Gartner et al., 2012; Racine et al., 2015). For example, a qualitative study found that the brain disease model is integrated into compassionate care but nevertheless risks downplaying the autonomy of those with an addiction (Szott, 2015).

A similar interpretation could help make sense of our findings about FW_VOLITION_. We found that increasing neuroscience-knowledge resulted in increasing attribution of FW_VOLITION_ (alcohol and cocaine) in the control group which perhaps reflects other findings suggesting that higher education is associated with increased beliefs in volition and the ability for self-determination or the sheer valuing of autonomy (Ryan and Deci, 2000; Say et al., 2006; Zizzo et al., 2017). However, we found no such trend in the other treatments. Results for these other treatments suggest that some of them (only consistently shown in the *neuroimage-only-*treatment) partially undermine higher attribution of FW_VOLITION_ associated with increased neuroscience-knowledge. Perhaps neuroscience information induces a deterministic view that diminishes attribution of FW_VOLITION_ in those with greater knowledge of neuroscience just like it reduces attribution of responsibility. These effects would fit in the broader context where both academic (Saigle et al., 2017) and public discussions (Racine et al., 2017) of neuroscience research about volition tend to be casted in deterministic overtones such that the actual existence of free will is seriously questioned.

In sum, these interaction effects between self-reported knowledge of neuroscience and belief in free will are intriguing even if a comparison with previously published results is difficult because of the different measures used to capture constructs such as level of education or level of neuroscience knowledge (e.g., self-reported knowledge of neuroscience in our study, level of advancement in a graduate program for McCabe and Castel). Nonetheless, these interactions suggest that the study of a phenomenon like the effects of neuroscience explanations is complex and needs to carefully integrate information about the context in which the prompting information is introduced (e.g., addiction, substance, or another context) and about the person considering this information (e.g., based on respondent characteristics such as neuroscience knowledge). Our observations also support the need for new, more refined scales to clearly tease apart the constructs of volition and responsibility, such as the Free Will Inventory which specifically recognizes this problem and proposes a cleaner measure of free will *per se* (separated from moral responsibility) as well as the ability to examine the relationships between these important constructs (Nadelhoffer et al., 2014).

## Conclusion

There has been much research debating the impact of neuroscience information generally and particularly in the discussion about the merits and drawbacks of a brain disease model of addiction. Within this context, the impact of neuroscience information on belief in free will is of particular interest because of its alleged impact on stigma and attitudes toward responsibility and treatment. In one of the largest studies undertaken thus far, we found no evidence that plausible textual neuroscience information impacted belief in free will (research question 1), which was subsequently divided into two factors: volition and responsibility. Likewise, neuroimaging, considered to potentially be highly persuasive in the literature, had no impact (research question 2). We only found that neuroimages in combination with textual information slightly lowered FW_VOLITION_ attributed to people with a cocaine addiction (research question 3). In addition, we found that FW_RESPONSIBILITY_ attribution for people with alcohol addiction are lower in the *neuroimage*-*only*-treatment compared to the textual information. Several respondent characteristics weakly co-varied with attribution of volition and responsibility (research question 4). Interaction analyses revealed that only neuroscience-knowledge interacted with different treatments (research question 5), thus calling for greater attention to the effects of such respondent characteristics. Overall, the concerns underlying the literature on the seductive allure of neuroscience as well as on the (positive or negative) effects of the brain disease model of addiction could be overdone, although this would merit more precise investigations and replication. In this vein, we recommend that future research pays more attention to the plausibility of the treatments used, since strongly-worded treatments may induce significant effects that more moderate, but perhaps more ecologically plausible treatments do not. Finally, possible interaction effects between treatments and respondent characteristics should be more carefully considered and investigated, as well as new instruments that are specifically designed to disentangle the factors of volition and responsibility often captured under the single construct of free will.

## Author contributions

AE and SS designed the study and wrote the protocol with ER. ER performed literature searches along with AE and SS. SS conducted statistical analyses and drafted the results section and AE and ER provided additional comments on data interpretation and presentation. ER drafted other sections of the manuscript and SS and AE provided substantive comments and made changes. All authors contributed to drafting the work and revising it critically for important intellectual content. All authors approved the final version of the manuscript and all agree to be accountable for all aspects of the work.

### Conflict of interest statement

The authors declare that the research was conducted in the absence of any commercial or financial relationships that could be construed as a potential conflict of interest. The reviewer HC and handling Editor declared their shared affiliation, and the handling Editor states that the process nevertheless met the standards of a fair and objective review.

## Footnotes

^1^NIDA. *Commonly Abused Drug Chart*. Last modified January, 2016. Available online at: http://www.drugabuse.gov/drugs-abuse/commonly-abused-drugs/commonly-abused-drugs-chart

^2^For this procedure, we first calculated the base-line reading speed (BLRS) of each respondent. This was measured by the response time to two education questions (highest high school degree and the highest vocational training qualification or university/college degree), since they assess simple facts. Generally, those respondents for which a response time could not accurately assessed (i.e., respondents went back and forth on the relevant questionnaire pages) had to be excluded from our analysis. Furthermore, to reduce measurement errors for the BLRS, measures below the 1 percentile (e.g., responses of less than 4 s) and those above the 99% percentile (e.g., responses of more than 163 s) were excluded. Then, (1) each of the two response times was z-standardized, (2) both were averaged, and (3) this mean score was again z-standardized. With the BLRS, individual exposure times to our treatments were predicted in each treatment. This predicted exposure time was compared to the real exposure time and respondents. If respondents were exposed less than 33.3% of the expected time, they were excluded (e.g., expected time 37 s, but real exposure 10 s). According to that procedure, 29 respondents were identified as being too fast in the *text-only-*treatment, 38 in the *text and neuroimage*-treatment, and 35 in the *neuroimage-only*-treatment.

^3^Brain Facts. *Addiction: Introduction. Disease and Disorders*. http://www.brainfacts.org/diseases-disorders/addiction/ (Accessed March 17).

^4^One exception is an interaction effect between the *text and neuroimage*-treatment and peer SUD (*p* = 0.027), indicating that respondents knowing peers with cocaine SUD had the lowest attributed FW_VOLITION_ of people with addiction to cocaine. Due to the singularity of this interaction and given the relatively low prevalence of peer SUD, we do not further discuss this finding, but we encourage forthcoming research to do so.

^5^As our interaction analyses do not allow a clear interpretation whether neuroscience-knowledge moderates the effect of the experimental treatments or whether the experimental treatments moderates the effect of neuroscience-knowledge, this and the following presentation of the interactions effects could also be reversely described, e.g., this interaction effect could be also described as: Presenting a neuroimage results in a lower attributed FW_VOLITION_ regarding people with addiction to alcohol with increasing neuroscience-knowledge.
